# Transcranial Direct Current Stimulation Combined With Meditation for Older Adults With Knee Osteoarthritis

**DOI:** 10.1093/geroni/igaa057.663

**Published:** 2020-12-16

**Authors:** Hyochol Ahn, Lindsey Park, Hongyu Miao

**Affiliations:** University of Texas Health Science Center at Houston, Houston, Texas, United States

## Abstract

Osteoarthritis (OA) of the knee is one of the most common causes of pain in older adults. Recent evidence suggests that knee OA pain is characterized by alterations in central pain processing in the brain. Two nonpharmacological pain treatments, transcranial direct current stimulation (tDCS) and mindfulness-based meditation (MBM), have been shown to improve pain-related brain function in older adults with knee OA. Because tDCS promotes neuroplasticity, it may potentiate the effect of MBM that also stimulates adaptive changes in the brain. However, no studies have examined whether tDCS combined with MBM can reduce OA symptoms in older adults with knee OA. Thus, the purpose of this study was to examine the preliminary efficacy of tDCS combined with MBM in older adults with knee OA. Thirty participants with knee OA were randomly assigned to receive 10 daily sessions of home-based 2 mA tDCS combined with active MBM for 20 minutes (n=15) or sham tDCS combined with sham MBM (n=15). We measured OA-related clinical symptoms using the Western Ontario and McMaster Universities Osteoarthritis Index (WOMAC). Participants (60% female) had a mean age of 59 years. Active tDCS combined with active MBM significantly reduced scores on the WOAMC (Cohen’s d = 0.83, P = 0.02). Participants tolerated tDCS combined with MBM well without serious adverse effects. Our findings demonstrate promising clinical efficacy of home-based tDCS combined with MBM for older adults with knee OA. Future studies with larger-scale randomized controlled trials with follow-up assessments are needed to validate our findings.

